# Biochemical profile of bile fluid in patients with malignant cholestasis in comparison with cholestasis due to gall stone 

**Published:** 2015

**Authors:** Hassan Taheri, Naser Ghemian, Yaser Taghavi, Javad Shokry-Shirani

**Affiliations:** 1 Department of Internal Medicine, Babol University of Medical Sciences, Babol, Iran.; 2 Department of Radiology, Babol University of Medical Sciences, Babol Iran.

**Keywords:** Cholangiocarcinoma, Tumor marker, Biochemical profile

## Abstract

**Background:**

Cholangiocarcinoma is an invasive biliary malignancy with poor prognosis. Diagnostic accuracy of conventional methods is low which is mainly due to the specific anatomy of the disease. The aim of this study was to evaluate the diagnostic value of biochemical profile and tumor marker of the bile in patients with malignant cholestasis compared to that of choledocholithiasis.

**Methods:**

In this cross-sectional study, 46 patients with extrahepatic cholestasis were enrolled (20 patients with malignant cholestasis and 26 patients with choledocolithiasis) A definitive diagnosis of cholangiocarcinoma was made by imaging, cytology and biopsy. Bile fluid was obtained by aspiration through endoscopic retrograde cholagiopantreatography (ERCP) catheter or percutaneous drainage in patients with choledocolithiasis and cholangiocarcinoma respectively. Sex and age were matched in two groups. Data regarding the biochemical profile (triglyceride, (TG), cholesterol, billirubin and HDL) and CA19.9 level of the bile fluid were collected, then using the SPSS software, the data were analyzed.

**Results:**

Bile fluid level of TG, cholesterol, high – density lipoprotein (HDL), direct bilirubin and CA19.9 were significantly higher in patients with benign cholestasis in comparison with malignant cholestasis (P<0.001, P<0.001, P<0.001, P=0.012 and P= 0.03, respectively).

**Conclusion:**

Our study showed that the CA19.9 level of bile fluid in extrahepatic cholestasis due to biliary stone was significantly higher than those with cholangiocarcinoma, as is the biliary level of TG, cholesterol, high-density lipoprotein (HDL) and direct bilirubin. Thus they may help in the differentiation of benign versus malignant extra hepatic cholestasis.

**C**hlangiocarcinomas arise from the epithelial cells of the bile ducts. These cancers are highly lethal because mostly are locally advanced at presentation. Due to the specific anatomy of the disease, access to the tumor for tissue sampling is difficult and imaging methods such as endoscopic retrograde chollangiopanereatography (ERCP) with brushing have less than 50% yield at the best (1, 2). Cholangiography performed by ERCP or via a percutaneous approach is the gold standard for defining the location and extension of suspected bile duct lesion but up to one third of the patients with symptoms and cholangiogram suggestive of a bile duct malignancy will have either benign fibrosing disease or another malignancy with metastases that obstruct the bile ducts.

Tissue diagnosis can be obtained by a variety of means in patients suspected of having a hilar malignancy but is often difficult to obtain. Endoscopic brush cytology has a limited sensitivity and the addition of endoscopic biopsy of the malignant strictures increases this sensitivity up to 60%. Therefore diagnostic methods using biochemical and molecular markers which are present in the bile fluid and have a higher sensitivity for the diagnosis of cholangiocarcinoma may be used for this purpose. 

In the recent years, it has been attempted to differentiate malignant from non-malignant extrahepatic cholestasis by using biochemical and cytologic markers of the bile fluid. O’Mahony et al. studied molecular mutations in the bile samples of patients with pancreatic and biliary cancers (3). Serum levels of cancer antigen CA19.9 are widely used for detecting cholangiocarcinoma but optimal cutoff value that best discriminates between benign or malignant biliary disease is influenced by the presence of cholangitis or cholestasis (4). In other studies, it has been tried to differentiate between malignant and non-malignant biliary disease using FNA assisted cytologic evaluation (5, 6).

This study was designed to evaluate the diagnostic value of biochemical profile and tumor marker level of bile fluid in patients with malignant cholestasis in comparison with cholestasis due to gall stone.

## Methods

In a 2-year period (between March 2008 and July 2010), twenty patients diagnosed with cholangiocarcinoma were enrolled in this study, then these patients were compared with a control group of 26 patients with choledocholithiasis and symptoms of cholangitis undergoing endoscopic therapy for stone removal. The patients with pancreatic cancer were excluded from the study. This study was approved by the Ethics Committee of Babol University of Medical Sciences, an informed consent was taken from all patients before the study. In patients with cholangiocarcinoma, 10 cc of bile fluid was obtained during percutaneous bile drainage while inserting drainage catheter for palliative therapy and in patients with choledocolithiasis, 10 cc of bile fluid was collected through ERCP catheter during common bile duct (CBD). cannulation. The collected bile sample was then sent to the hospital laboratory department for storage and future analysis of the biochemical and molecular markers. The variables studied were triglyceride (TG), cholesterol (Chol), high density lipoprotein (HDL), total and direct bilirubin levels (T Bil and D Bil) and CA19.9.

For the evaluation of the biochemical variable photometric method was used (Pars Azmoon Co, kits) and for assessing the CA19.9 of bile, Elisa method was used (Fuji Roberto Co, Sweden). All the patients’ blood was taken for serum level of aspartate aminotransferase (AST), alanine aminotransferase (ALT), alkaline phosphatase (AlkPhos) and bilirubin (bili) for comparison. Then we compared the demographic and biochemical variables of bile fluid in two groups. Chi-Square test was used for categorical variables and t-test or Mann-Whitney U-test for continuous variables, SPSS (Windows 16) was used for statistical analysis, p-value of less than 0.05 was considered significant. For the evaluation of sensitivity and specificity, ROC curve was used.

## Results

A total of 20 patients with cholangiocarcinoma (group 1) were compared with 26 patients with choledocolithiasis (group 2). Twelve (65%) of patients in group 1 and 14 patients (52.8 %) in group 2 were men (P=0.44). The mean age of patients in group 1 was 66±10 years and the mean age of patients with choledocolithiasis was 55.1±18 years, (P=0.07). Serum ALT and AST levels were higher in group 2 while alkaline phosphatase (ALP) and total bilirubin levels were higher in group 1, respectively. Blood level of direct bilirubin was significantly higher in group 2, however (table 1). Bile fluid composition was compared in 2 groups (table 2). 

**Table 1:** Comparison of serum biochemical factors in two group of patients

**Serum biochemical factors**	**Malignant cholestasis**	**Stone induced cholestasis**	**P value**
AST(IU/L)	87.00±23.06	167.67±36.15	0.169
ALT(IU/L)	91.87±32.23	242.00±46.01	0.050
AlkPhos (IU/L)	1269.88±335.91	852.50±204.53	0.282
T Bili (mg/dl)	11.60±2.72	5.47±1.63	0.054
D Bili (mg/dl)	5.91±1.58	2.27±0.85	0.006

The concentration of TG, cholesterol, HDL, direct bilirubin and CA19.9 was significantly higher in patients with gall stone compared with those who had cholangiocarcinoma. Table 3 shows the positive and negative predictive values (PPV and NPV) of these biochemical and molecular factors for cholestasis due to gall stones.

**Table 2:** Comparison of bile fluid biochemical factors in two groups of patients

**Bile fluid biochemical factors**	**Malignant cholestasis**	**Stone induced cholestasis**	**P-value**
Triglyceride(mg/dl)	21.40±2.15	43.58±5.47	0.001
Cholesterol(mg/dl)	28.90±5.06	58.42±7.68	0.001
LDL(mg/dl)	15.80±4.41	27.00±5.62	0.142
HDL(mg/dl)	6.85±0.79	27.46±3.12	0.000
T Bili (mg/dl)	19.70±8.06	36.81±4.89	0.064
D Bili (mg/dl)	2.85±1.15	7.57±1.30	0.012
CA19.9(U/ml)	325.05±88.75	905.25±421.63	0.030

**Table 3:** Comparison of sensitivity, specificity, positive and negative predictive value of bile fluid biochemical factors for stone-induced cholestasis

**Bile fluid biochemical factors**	**Sensitivity (%)**	**Specificity (%)**	**PPV (%)**	**NPV (%)**
Triglyceride (29.5 mg/dl)	69	90	90	69
Cholesterol (18.5 mg/dl)	92	30	63	75
HDL(9mg/dl)	96	80	86	94
D Bili (6.45 mg/dl)	46	95	92	57
CA 19.9 (111 U/ml)	100	40	17	83

## Discussion

Our study showed that bile fluid TG and cholesterol were higher in subjects with choledocolithiasis in comparison with malignant obstruction of bile duct. Mean TG concentration of bile fluid was 43.5±5.4 mg/dl and 21.4±2.1 mg/dl (P<0.001) in subjects with biliary stone and cholangiocarcinoma, respectively. TG level above 29.5 mg/dl showed 69% sensitivity, 90% specificity, 90% PPV and 69% NPV for stone-induced cholestasis. Bile cholesterol concentration was 58.4±7.6 mg/dl and 28.9±5 mg/dl (P<0.001) in patients with choledocolithiasis and cholangiocarcinoma, respectively. Bile fluid total cholesterol level above 18.5 mg/dl showed 92% sensitivity, 30% specificity, 63% PPV and 75% NPV for stone-induced cholestasis. 

The HDL concentration of bile was 27.4±3.1 mg/dl and 6.8±0.8 mg/dl (P<0.001) in patients with bile duct stone and cholangiocarcinoma, respectively. An HDL level above 9 mg/dl had 96% sensitivity, 80% specificity, 86% PPV and 94% NPV for the diagnosis of stone-induced cholestasis.

There are few studies concentrating on lipid component composition of bile. Gustafsson et al. performed a study on total lipid component of bile in subjects with and without gall stones, they found elevated cholesterol levels in patients with gall stones. Total lipid (TG, cholesterol and phospholipid) levels was 9±0.4mg/dl and 12.9±0.5 mg/dl in those with and without gall stones, respectively which is statistically significant, but cholesterol level of bile was 7.8±0.2 mol % and 5.4±0.2 mol% in those with and without gall stones, respectively (7). We could not find any study which compares the lipid composition of bile in those with gall stones and cholangiocarcinoma. This difference in the composition of bile fluid lipid component could be due to fixed obstruction of the bile ducts in cholangiocarcinoma which caused diminished expression of some export proteins.

Total and direct bilirubin level of bile fluid in these two groups was assessed in our study, there was no significant difference in the total bilirubin level of bile fluid in two groups which was 19.7±8.0 mg/dl in cholangiocarcinoma and 36.81±4.8 mg/dl in the gall stone group (P=0.064). But the difference in the level of direct bilirubin was statistically significant which was 2.8±1.1 mg/dl in cholangiocarcinoma and 7.5±1.3 mg/dl in gall stone group (P=0.012). A direct bilirubin level of the bile fluid above 6.45 mg/dl had 46% sensitivity, 95% specificity, 92% PPV and 57% NPV for the diagnosis of stone induced cholestasis. In normal state the main bilirubin of bile fluid is direct bilirubin, the proposed reason for the difference seen in patients with bile duct cancer is that chronic cholestasis may alter the conjugation capacity of hepatocytes.

In our study, we analyzed the serum level of AST, ALT, and ALP in two groups. We found higher level of transaminases in patients with choledocolithiasis, in stone-induced obstruction an acute elevation of the intrahepatic bile duct pressure is produced that leads to abrupt elevation of transaminases but in malignant biliary obstruction, this elevation is blunted and high concentration of transaminases is not seen.

Serum level of AlkPhos was higher in patients with malignant obstruction as was the level of total and direct serum bilirubin. Qin et al. found a higher level of serum ALP in those with malignant biliary tract disease versus benign biliary tract disease, but the serum level of AST was higher in patients with malignant biliary disease in their study which is in contrast with our study (8).

In our study, bile fluid level of CA19.9 was 325±88 U/ml in patients with cholangiocarcinoma and 905±421 U/ml in those with stone-induced cholestasis (P= 0.03). CA19.9 level above 111 U/ml had 100% sensitivity, 40% specificity, 17% PPV and 83% NPV for the diagnosis of stone-induced biliary obstruction. Akdogan et al. in a study which was done on the serum and bile fluid level of CA19.9 in patients with malignant and stone induced biliary obstruction found a mean bile fluid CA19.9 level of 14000 and 14818 U/ml in those with malignant and stone-induced biliary obstruction, respectively (P=0.8). 

The serum CA19.9 level was higher in malignant group in their study, however (9). Chen et al. performed a study on the sensitivity and specificity of serum CA19.9 level in those with cholangiocarcinoma, in their study a serum CA19.9 level of 20000U/ml had 52% sensitivity,60% specificity, and 37% PPV (10). The same result was seen in Lindberg et al.’s study (11). 

In Ohshio et al.’s study there was no significant difference in the bile fluid CA19.9 level between those with malignant compared to non-malignant disease (12). Peterli, Murohisa and Sheen Chen showed that in stone-induced cholangitis serum CA19.9 level may reach to values as high as 60000 U/ml. Mann et al. in their study showed that only half of the patients increased serum CA19.9 level eventually had malignancy (13-16).

In conclusion, we showed that the measurement of bile fluid lipid profile especially TG , HDL and its direct bilirubin and CA 19.9 level may be a useful complementary test in differentiation between benign and malignant cholestasis. 

## Notes

**Funding: **This study is a thesis project of Yaser Taghavi (No: 305).
